# A context‐specific conceptual framework of evidence synthesis to improve childhood cancer health outcomes and resource use in Egypt: Using real‐world data and addressing the implementation gaps

**DOI:** 10.1002/cesm.12010

**Published:** 2023-04-17

**Authors:** Ranin Soliman

**Affiliations:** ^1^ Department of Continuing Education, Centre for Evidence‐Based Medicine University of Oxford Oxford UK; ^2^ Health Economics and Value Unit Children's Cancer Hospital Egypt (CCHE) Cairo Egypt

**Keywords:** childhood cancer, conceptual framework, Egypt, evidence synthesis, real‐world data, resource‐limited settings

## Abstract

**Introduction:**

Given the large numbers of children with cancer in Egypt, the limited resources, and inferior survival outcomes, there is a need to better target resources to improve outcomes efficiently based on evidence. Nevertheless, there is a gap in knowledge about childhood cancer health outcomes and resource use in Egypt. This commentary presents a “context‐specific” conceptual framework of evidence synthesis to improve childhood cancer health outcomes and resource use in a resource‐limited setting in Egypt, using real‐world data and addressing the implementation gaps.

**Methods:**

Real‐world data is defined as data relating to health status and/or the delivery of health services routinely collected from various sources outside the contexts of randomized controlled trials that can be used to conduct prospective/retrospective observational research studies.

**Results:**

To better address this context‐specific clinical problem, the conceptual framework of evidence synthesis proposes to generate three types of evidence using hybrid research methods; (1) Real‐world evidence (obtained from observational studies based on routinely collected data from local context); (2) systematic evidence from the literature (systematic review); and (3) qualitative evidence based on experts' opinions in the local setting (interview study). Generating evidence from the three pillars altogether makes for a stronger approach to better research and tackle the local problem in this specific resource‐limited context, and address the implementation gaps.

**Conclusions:**

This framework serves as a methodological roadmap to generate relevant evidence in similar resource‐limited contexts in low‐ and middle‐income countries, where there is a paucity of published studies in the literature about childhood cancer survival outcomes and resource use.

## 1 INTRODUCTION

Childhood cancer is a major cause of global disease burden, where around 80% of this burden occurring in low‐ and middle‐income countries (LMICs) [1, 2]. Hence, childhood cancer in LMICs is a global child health priority, due to the large proportions of children with cancer, limited resources, and inferior survival [3]. The WHO *Global Initiative of Childhood Cancer* was launched to increase the global childhood cancer survival to at least 60% by 2030 [4], focusing on improving the much lower survival in LMICs to address the inequalities in care and outcomes [4].

## 2 CHILDHOOD CANCER AS A HEALTH PRIORITY IN EGYPT

Egypt is a lower middle‐income country that ranks tenth globally with the greatest numbers of incident childhood cancer cases [5]. This is attributed to the large population of Egypt [6], of which 34% are children under the age of 15 [7]. The 5‐year survival of childhood cancer in Egypt was estimated to be around 40% based on modeled data [8], compared to over 80% in high‐income countries [9]. Childhood cancer treatment is resource intensive and costly, and it is difficult to accommodate the high demand for services due to the limited resources [8, 10]. Accordingly, childhood cancer is a health priority in Egypt, and there is an urgent need to better target the available resources to improve care and survival outcomes efficiently. However, there is a paucity of childhood cancer survival data in Egypt, due to absence of survival data in population‐based cancer registries [11], and limited published litearture [12]. Moreover, no local data exist on resource use/costs and treatment cost‐effectiveness [13]. Therefore, it is crucial to generate high‐quality evidence to improve childhood cancer health outcomes and resource use in Egypt.

## 3 CONCEPTUAL FRAMEWORK OF EVIDENCE SYNTHESIS

This commentary presents a context‐specific conceptual framework of evidence synthesis to improve childhood cancer health outcomes and resource use in Egypt, using real‐world data and addressing the implementation gaps. A conceptual framework is a set of interlinked key concepts, factors, or variables that together provide a comprehensive understanding of a phenomenon of interest, where each concept plays an integral role [14].

Just as different locks need different keys to unlock them, different problems need different solutions to address them. The conceptual framework builds upon three key concepts to better address this context‐specific problem: conducting primary research from local context to address the gap in literature; conducting systematic review as the main gold standard for evidence synthesis; and addressing the implementation gaps. The conceptual framework was also inspired by key concepts in evidence‐based medicine (EBM) (Box 1) [15, 16, 17].

Box 1 **Key EBM concepts that inspired the conceptual framework**

- Different types of research questions require different types of evidence [15].
- Generating evidence that is relevant to clinical practice and applies to our patients [16].
- EBM is about integrating individual clinical expertise and the best external evidence [17].
- Irrespective of the type of research, systematic reviews are necessary [15].

**Figure 1:**
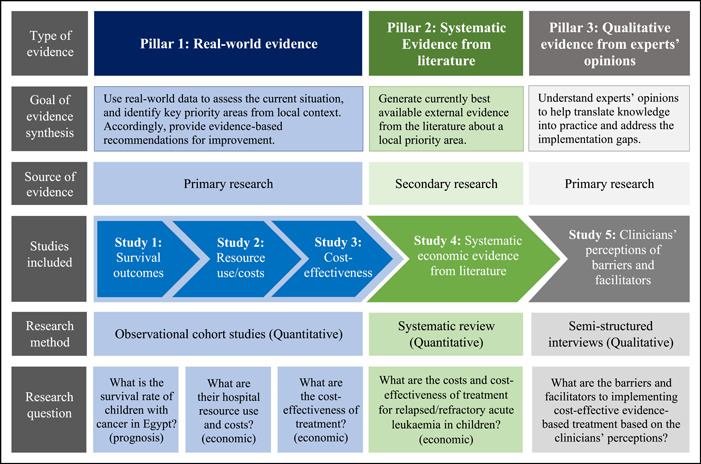
Conceptual framework of evidence synthesis to improve childhood cancer health outcomes and resource use in Egypt.

In light with the above, the conceptual framework of evidence synthesis (Figure 1) proposes to generate three types of evidence using hybrid research methods: (1) Real‐world evidence (observational cohort studies based on routinely collected data from local context); (2) systematic evidence from the literature (systematic review); and (3) qualitative evidence based on experts' opinions from local context (interview study). In this context‐specific conceptual framework, the term “*evidence synthesis*” refers to generating the three different types of evidence altogether, rather than the more commonly used/known term that only refers to systematic reviews. This conceptual framework was implemented at the Children's Cancer Hospital Egypt (CCHE); a non‐profit pediatric oncology setting that treats children with cancer for free based on philanthropic donations [18].

### 3.1 Pillar 1: Real‐world evidence from local context

The first pillar of the conceptual framework recommends using real‐world data from local context to generate relevant evidence. Real‐world data is defined as data relating to patient health status and/or the delivery of health services routinely collected (outside the context of randomized controlled trials) from various data sources including: electronic health records, disease registries, or administrative/costing databases. Real‐world data can be used to conduct observational cohort research studies, either prospectively or retrospectively [19]. Real‐world evidence is defined as the evidence generated from the careful analysis and interpretation of real‐world data [19, 20]. To retrieve and use/analyze local routinely collected data, approval from the scientific medical advisory committee is required, whereas ethical consent is not necessary. Generating real‐world evidence helps assess the current situation at the local context and identify any problems in care and/or outcomes of treatment as key priority areas for improvement. Accordingly, this helps provide evidence‐based recommendations to improve outcomes and inform clinical and policy decisions.

#### 3.1.1 Childhood cancer survival

The first step in the conceptual framework is to generate real‐world evidence about childhood cancer survival in Egypt/CCHE through conducting a retrospective observational cohort study [study 1]. This prognostic study uses routinely collected demographic, disease‐related, and survival data extracted from the hospital‐based cancer registry at CCHE/Egypt. This study identifies the childhood cancer types with inferior survival (over time), and help provide evidence‐based recommendations to improve survival and reduce the existing inequalities in care and outcomes between this LMIC‐context and other developed countries [21]. Therefore, helps make informed clinical decisions to modify practice/treatment for cancers with poor outcomes. Full‐text of this study is available at: https://onlinelibrary.wiley.com/doi/full/10.1002/ijc.33321.

#### 3.1.2 Childhood cancer resource use and costs

The second study is a retrospective observational cohort study aiming to determine childhood cancer hospital resource use (frequency of hospital admissions/length‐of‐stay; numbers of investigations/treatment interventions) and costs of care at CCHE/Egypt [study 2]. This study uses resources use data retrieved from electronic medical records, and costs data retrieved from the administrative/costing database, to generate economic evidence from the local context through determining hospital resource use and associated costs [22]. Therefore, findings from this study identify cancers with highest resource use/costs, and provide evidence‐based recommendations that can help inform policy decisions to optimize resource use and manage costs effectively. Full‐text of this study is available at: https://onlinelibrary.wiley.com/doi/10.1002/pbc.29347.

#### 3.1.3 Cost‐effectiveness of treatment

This study uses a retrospective observational cohort study design to analyze routinely collected survival outcomes and costs, which are then used to estimate cost‐effectiveness in terms of cost per disability‐adjusted life‐years averted (a GDP‐based threshold). [study 3]. This study generates economic evidence from the local context through estimating cost‐effectiveness of childhood cancer treatment [22]. Findings from this study helped identify childhood cancer types/groups (i.e., relapsed/refractory acute leukemia) whose treatment was not cost‐effective, indicating a significant local problem and a key priority area for improvement. This shall help make informed policy and clinical decisions to promote treatment cost‐effectiveness. Full‐text of this study is available at: https://www.thelancet.com/journals/eclinm/article/PIIS2589-5370(22)00459-X/fulltext.

### 3.2 Pillar 2: Systematic evidence from the literature

The second pillar of the conceptual framework recommends generating systematic evidence from the literature to address a local problem/priority area identified from the previous study Therefore, this study generates economic evidence through conducting a systematic review of costs/cost‐effectiveness of treatment for relapsed/refractory pediatric acute leukemia [study 4] [22]. Systematic reviews seek to answer a specific research question by summarizing evidence aiming to minimize bias by using explicit, and systematic methods [23]. In this context, the systematic review summarises the currently best available evidence about cost‐effective therapies that can help clinicians and policy‐makers make better informed clinical/policy decisions to explore/adopt more cost‐effective strategies. Full‐text of this study is available at: https://www.tandfonline.com/doi/full/10.1080/17474086.2022.2069096.

### 3.3 Pillar 3: Qualitative evidence from local context

The third pillar of the conceptual framework recommends to generate qualitative evidence from experts' opinions on the barriers and facilitators to research implementation/translation at the local context. This would help address the implementation gaps and translate the generated evidence into practice [24]. This is a qualitative semi‐structured interview study [study 5] of the clinicians' perceptions of barriers and facilitators to implementing cost‐effective evidence‐based childhood cancer treatment in a resource‐limited context in Egypt. Findings from this study can inform clinical and policy decisions to overcome the existing barriers and develop tailored strategies and practical mechanisms for implementation.

## 4 STRENGTHS AND LIMITATIONS

Evidence synthesis from all three pillars of the framework altogether makes for a stronger approach to better research and tackle the local problem for this specific population in resource‐limited LMIC contexts. Therefore, this conceptual framework can serve as a methodological roadmap to generate high‐quality evidence to improve childhood cancer health outcomes and resource use in similar resource‐limited settings in Egypt or other LMICs.

Despite the significance of this conceptual framework, some limitations exist. Generally, real‐world data studies can be limited by potential biases during study design or result interpretation; heterogeneity in quality of real‐world data; and limited generalizability of results [20, 25]. Limitations at our local setting included inadequate cost data infrastructure, and predominance of the hospital's context and culture based on the available diagnostic and treatment infrastructures.

## 5 THE WAY FORWARD TO ADDRESSING THE IMPLEMENTATION GAPS

Ideally, experts' opinions of other key stakeholders should also be considered in future work such as consumers (patients/families) and payers (decision‐makers in the fundraising foundation that collects donations to treat children with cancer at CCHE). This framework should be integrated with knowledge translation work to implement the generated research‐based knowledge into practice [26]. However, LMICs face many barriers to translate knowledge into practice due to limited resources, social/cultural contexts, and resistance to change [26]. The Ottawa Model of Research Use provides a helpful approach for knowledge translation, which is adaptable to LMIC‐contexts; it begins with assessing the barriers/facilitators within the practice environment and suggests tailoring the knowledge translation strategies accordingly [26, 27]. This is aligned with the third pillar of the framework, which provides context‐specific suggestions to overcome existing barriers, and recommends practical implementation mechanisms.

## CONFLICT OF INTEREST STATEMENT

Ranin Soliman is supported by Egypt Cancer Network (ECN) to develop this conceptual framework and conduct the underlying research studies, as part of her DPhil in Evidence‐based Healthcare (EBHC) at the University of Oxford, UK. The funder had no role in the design, conduct, or publication of the content, research, or evidence synthesis. No other conflicts of interest exist.

## Data Availability

Data sharing is not applicable to this article as no new data were created or analyzed in this study.
